# Common Variable Immunodeficiency Disorders as a Model for Assessing COVID-19 Vaccine Responses in Immunocompromised Patients

**DOI:** 10.3389/fimmu.2021.798389

**Published:** 2022-01-18

**Authors:** Rohan Ameratunga, See-Tarn Woon, Richard Steele, Klaus Lehnert, Euphemia Leung, Emily S. J. Edwards, Anna E. S. Brooks

**Affiliations:** ^1^ Department of Clinical Immunology, Auckland Hospital, Auckland, New Zealand; ^2^ Department of Virology and Immunology, Auckland Hospital, Auckland, New Zealand; ^3^ Department of Molecular Medicine and Pathology, School of Medicine, Faculty of Medical and Health Sciences, University of Auckland, Auckland, New Zealand; ^4^ Department of Respiratory Medicine, Wellington Hospital, Wellington, New Zealand; ^5^ School of Biological Sciences, University of Auckland, Auckland, New Zealand; ^6^ Maurice Wilkins Centre, School of Biological Sciences, University of Auckland, Auckland, New Zealand; ^7^ Auckland Cancer Society Research Centre, School of Medicine, Faculty of Medical and Health Sciences, University of Auckland, Auckland, New Zealand; ^8^ Allergy and Clinical Immunology Laboratory, Department of Immunology and Pathology, Central Clinical School, Monash University, Melbourne, VIC, Australia

**Keywords:** COVID-19, SARS-CoV-2, CVID - common variable immunodeficiency disorders, vaccine responses, antibody deficiency

## Introduction

COVID-19 has had a disastrous impact on the world with over 5 million deaths, hundreds of millions infected and many more plunged into poverty. COVID-19 has affected almost all countries. The origin of the virus is the subject of ongoing study (1–3).

SARS-CoV-2 initially infects the nasal mucosa. The Spike (S) glycoprotein engages cell-surface ACE2. Host proteases including TMPRSS-2 cleave the S glycoprotein, allowing the S2 subunit to fuse with the cell membrane (4). The viral genome is then able to hijack cellular organelles leading to production of daughter virus particles.

In the initial asymptomatic nasal phase, the innate immune system is silenced resulting in an exponential increase in viral progeny. SARS-CoV-2 deploys several mechanisms to evade cytoplasmic viral sensors. Following the nasal phase, the virus infects the lungs, probably by microaspiration from the nasopharynx and stomach (5, 6). Patients suffering pneumonitis experience increasing dyspnoea and have elevated inflammatory markers.

The smaller percentage entering the systemic phase suffer acute respiratory distress syndrome (ARDS) and multiple organ dysfunction. Increased d-dimers indicate a risk of arterial and venous thromboembolic disease. In spite of invasive ventilation and extracorporeal membrane oxygenation, mortality remains very high in such patients.

## Patients at Increased Risk

There is a high case fatality rate in the elderly (7). In addition, patients with comorbidities including obesity, diabetes, hypertension, coronary artery disease, malignancy, renal and pulmonary disease are at increased risk of adverse outcomes (7–9). Patients of Black, Hispanic and South Asian origin also have a higher case fatality rate. Inequitable access to healthcare is at least partly responsible for these ethnic differences (10, 11).

Current data also suggests individuals with some immunodeficiency disorders are at increased risk of severe outcomes (12, 13). Patients with innate immune defects and T cell disorders are at greater risk than healthy individuals (14–16). In contrast, most studies indicate patients with X-linked agammaglobulinemia (XLA) without comorbidities appear to be at lower risk, inferring antibodies can in some circumstances be detrimental (17–20). Some authors are however less certain about the protective effect of XLA in COVID-19 outcomes (21, 22).

Immunocompromised patients are at risk of Chronic COVID-19, a dangerous stalemate between SARS-CoV-2 and impaired cellular immunity (23). Patients with Chronic COVID-19 can shed virus for months before either succumbing to or recovering from the infection. Such patients are vulnerable to intra-host viral evolution which could result in variants of high consequence (24). This is a public health emergency and prevention of Chronic COVID-19 is of the utmost priority.

## Response to COVID-19 Vaccines

**Figure 1:**
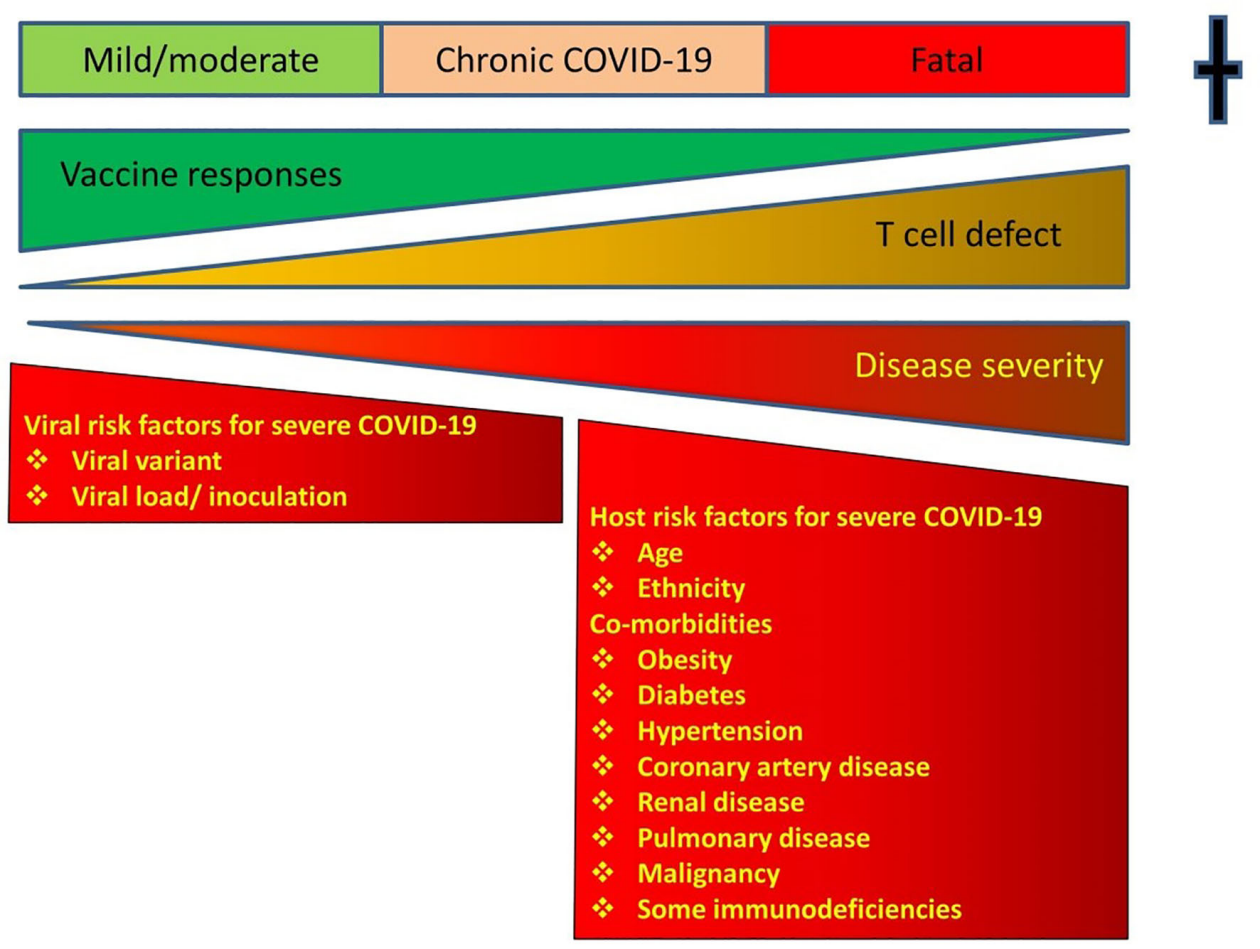
Showing the relationship between T cell defects, responses to vaccines and outcomes. Vaccines shift the disease severity to the milder end of the spectrum. Instead of fatal disease, vaccinated patients with T cell defects will have a milder version of the infection. There is a risk of Chronic COVID-19 in immunodeficient patients and a vigorous T cell response to vaccines shifts the disease severity to the milder end of the spectrum. COVID-19 disease severity according to WHO criteria.

Vaccines have proved effective in mitigating COVID-19. Vaccines do not prevent breakthrough infections, but markedly reduce the risk of a destructive immune response (**Figure 1**) (25). Hospitalisations and deaths from COVID-19 have dramatically decreased following vaccination. Most vaccinated patients dying from breakthrough infections are elderly or those with comorbidities. Vaccinated patients have variable levels of antibodies to the S glycoprotein at the time of breakthrough infections (26). There is no specific antibody level, which reliably prevents breakthrough infection (27, 28). The S glycoprotein is post-translationally modified with carbohydrates and antibody responses are less durable. In many studies antibody levels decrease six months after vaccination (29).

In contrast, memory T cell responses to vaccines correlate with protection (25, 30). Diagnostic T cell assays can be measured on different platforms, depending on the expertise of the laboratory (31). Current COVID-19 vaccines are regarded as T cell dependent and cellular responses are more durable, indicating that waning antibody levels underestimate the duration of protection (32). The S glycoprotein has strong adjuvant properties for cellular immunity, increasing its immunogenicity (and reactogenicity). Current COVID-19 vaccines do not require an additional adjuvant.

## Common Variable Immunodeficiency Disorders as a Model of Immunocompromised Individuals

Common Variable Immunodeficiency Disorders (CVID) are the most frequent symptomatic primary immune defect in adults and children (33, 34). By definition, the cause of CVID is not known (35–37). In some patients an underlying genetic defect is causative (38). If a causative defect is identified, these patients are considered to have a CVID-like disorder and are removed from the broad umbrella diagnosis of CVID. In non-consanguineous populations approximately 25% have an underlying genetic defect, mostly autosomal dominant disorders (39, 40). In consanguineous societies a much higher number have autosomal recessive disorders (41).

Currently there are three sets of diagnostic criteria for CVID in common use (35–37). The original European Society of Immunodeficiencies/Pan-American Group for Immunodeficiency (ESID/PAGID) 1999 Criteria required significant hypogammaglobulinemia (IgG 2 sd. below the mean) with either impaired vaccine responses or absent isohemagglutinins (42). These were deemed difficult to use (43). In 2013 new diagnostic criteria were proposed with a lower IgG threshold (5 g/L) and vaccine responses beyond protection, to those of normal persons (35). These criteria also contained many of the more recent discoveries including reduction in switched memory B cells and genes predisposing to CVID. In contrast to the previous criteria, impaired vaccine responses were not mandatory for the diagnosis. The revised ESID registry criteria were published in 2014 (36). These were very similar to the Ameratunga et al., criteria (35), but maintained the higher IgG threshold (2 sd. below the mean) and protective vaccine responses of the original ESID/PAGID 1999 Criteria. In 2016 the International Consensus (ICON) document was published (37). Like the original ESID/PAGID 1999 criteria, poor responses to vaccine were mandatory in the ICON 2016 Criteria.

CVID and CVID-like disorders have a spectrum of B and T cell defects. The ESID 2014 and ICON 2016 criteria exclude patients with severe T cell defects, who were deemed to have late onset combined immunodeficiency (LOCID) based on reduced naïve CD4^+^ T cell proportions (<10% CD4^+^ T cells) (36, 37). It has however been suggested patients with LOCID should remain within the broad spectrum of CVID and CVID-like disorders (44). Individuals within the same family, carrying the identical *NFKB1* mutation, had widely differing immune defects. One brother was in excellent health, while his sister suffered multiple autoimmune complications and malignancy. She met the criteria for LOCID because of reduced T cell subsets and died prematurely from hepatic failure (45, 46).

## CVID as a Model of Vaccine Challenge Responses in Immunocompromised Persons

Although not mandatory in the Ameratunga et al., 2013 or ESID 2014 Criteria, vaccine challenge responses are an integral part of the diagnostic work-up of patients with suspected CVID. CVID and other antibody deficiency disorders can serve as a useful model for both susceptibility to COVID-19 as well as responses to vaccines. In contrast to CVID, vaccine challenge responses are not routinely undertaken in patients suffering from secondary immunodeficiency disorders for either diagnosis or prerequisites for therapy. These patients receive subcutaneous or intravenous immunoglobulin (SCIG/IVIG) replacement based on either profound hypogammaglobulinemia or if they have modest hypogammaglobulinemia with breakthrough infections.

Two recent studies have explored the responses to vaccines in patients with hypogammaglobulinemia as well as CVID. In the New Zealand hypogammaglobulinemia study (NZHS), asymptomatic patients with hypogammaglobulinema (aHGUS) were noted to have an excellent prognosis (47). In this long-term prospective study, only one patient experienced progressive hypogammaglobulinemia requiring SCIG/IVIG. The majority have remained well for over a decade. In contrast, those with symptoms (sHGUS) had a mixed prognosis. Many experienced progressive deterioration culminating in SCIG/IVIG treatment. Vaccine challenge responses in the two groups were indistinguishable. Importantly, both groups had excellent responses to HIB and tetanus toxoid, both T cell dependent antigens. In contrast, responses to diphtheria toxoid and Pneumovax were muted. Poor responses to diphtheria toxoid are common, particularly in the elderly. Pneumovax responses are T cell independent.

A similar outcome was noted in the New Zealand CVID Study (NZCS) (48, 49). Most patients meeting criteria for CVID had excellent responses to tetanus toxoid and HIB. As in the NZHS, the responses to diphtheria toxoid and Pneumovax were suboptimal. This indicated T cell responses were preserved for at least some antigens in CVID. Recent studies confirm many CVID patients may generate protective responses to COVID-19 vaccines (50, 51).

## Approach to Immunocompromised Patients

The most important outcome of COVID-19 vaccination is a balanced, co-ordinated cellular immune response to the virus (25, 30). This implies at least some T cell function is required for vaccine efficacy (50). Given what was noted in the NZHS and NZCS, COVID-19 vaccines will provide at least partial protection in most immunocompromised patients. This is a strong argument for vaccinating these patients and monitoring their T cell responses to SARS-CoV-2 (50–52).

Immunocompromised patients should be individually assessed to determine the degree of cellular immune deficiency. The extent of cellular impairment can be ascertained by the types of infections as well as laboratory tests including T cell subsets and their *in vitro* function. The nature of the underlying disorder and therapy may also help identify impaired cellular immunity. Such an individualised approach can sometimes lead to unexpected findings. Patients treated with rituximab are more susceptible to COVID-19 than those with XLA (53–55). This may be because of the underlying disorder or because of the use of additional immunosuppressive agents.

The WHO, UK and other countries are now advocating a three dose primary COVID-19 immunisation program for immunocompromised persons. This may improve memory T cell responses to the vaccine (56, 57). It remains to be determined if heterologous primary immunisation, with mRNA and adenovirus-based vaccines generates a robust cellular response, as seen with humoral responses in healthy individuals (58). Again, monitoring T cell responses following vaccination will provide reassurance (50, 55).

It will be difficult to monitor antibody responses to COVID-19 vaccines if patients are on SCIG/IVIG. Most plasma donors have high titres of SARS-CoV-2 antibodies from either infection or vaccination (59). SARS-CoV-2 memory B cells could be quantified as a measure of humoral immunity in patients on IVIG/SCIG. These responses have been quantitated in PID patients receiving the influenza vaccine (60).

Antibody responses to the S glycoprotein are T cell dependent. In patients who are not on SCIG/IVIG, a good antibody response could be interpreted as a satisfactory cellular response to the vaccine. In those who have poor antibody responses, it is still possible they have protective T cell responses (50, 55). Many healthy persons failed to seroconvert but had robust T cell responses to SARS-CoV-2 (61). There have been calls for development of diagnostic T cell assays for SARS-CoV-2, which would be very useful for diagnosis or evaluating vaccine responses in immunocompromised patients (31, 61).

The best current advice is for immunocompromised patients including those with antibody deficiency to have at least three primary vaccinations and have their T cell responses measured (56, 57). If there is failure to generate cellular immunity to SARS-CoV-2, these patients should be advised to shelter in place until more effective therapeutics and vaccines are developed for COVID-19. The recent development of antiviral drugs by Merck (molnupiravir) and Pfizer (paxlovid) is encouraging. Until these drugs are widely available, patients with sub-optimal memory T cell responses remain at risk of severe outcomes or Chronic COVID-19 (**Figure 1**). If there is waning cellular immunity they should receive boosters. In the absence of a diagnostic T cell assay for SARS-CoV-2, booster COVID-19 vaccines could be routinely considered every 6 months or sooner.

## Author Contributions

RA wrote the first draft. All other authors contributed to editing the manuscript.

## Conflict of Interest

The authors declare that the research was conducted in the absence of any commercial or financial relationships that could be construed as a potential conflict of interest.

## Publisher’s Note

All claims expressed in this article are solely those of the authors and do not necessarily represent those of their affiliated organizations, or those of the publisher, the editors and the reviewers. Any product that may be evaluated in this article, or claim that may be made by its manufacturer, is not guaranteed or endorsed by the publisher.

## References

[1] Tiwari R Dhama K Sharun K Iqbal Yatoo M Malik YS Singh R. COVID-19: Animals, Veterinary and Zoonotic Links. Vet Q (2020) 40(1):169–82. doi: 10.1080/01652176.2020.1766725 PMC7755411 32393111

[2] Segreto RA Deigin Y. The Genetic Structure of SARS-CoV-2 Does Not Rule Out a Laboratory Origin: SARS-COV-2 Chimeric Structure and Furin Cleavage Site Might be the Result of Genetic Manipulation. Bioessays (2021) 43(3):e2000240. doi: 10.1002/bies.202000240 33200842 PMC7744920

[3] Holmes EC Goldstein SA Rasmussen AL Robertson DL Crits-Christoph A Wertheim JO. The Origins of SARS-CoV-2: A Critical Review. Cell (2021) 184(19):4848–56. doi: 10.1016/j.cell.2021.08.017 PMC8373617 34480864

[4] Hoffmann M Kleine-Weber H Schroeder S Kruger N Herrler T Erichsen S. SARS-CoV-2 Cell Entry Depends on ACE2 and TMPRSS2 and Is Blocked by a Clinically Proven Protease Inhibitor. Cell (2020) 4(20):30229–4. doi: 10.1016/j.cell.2020.02.052 PMC7102627 32142651

[5] Baek WK Sohn SY Mahgoub A Hage R. A Comprehensive Review of Severe Acute Respiratory Syndrome Coronavirus 2. Cureus (2020) 12(5):e7943. doi: 10.7759/cureus.7943 32499983 PMC7266564

[6] Ameratunga R Woon ST Steele R Snell R Medlicott N Mears E. Perspective: The Nose and the Stomach Play a Critical Role in the NZACE2-Pātari* (Modified ACE2) Drug Treatment Project of SARS-CoV-2 Infection. Expert Rev Clin Immunol (2021) 17(6):553–60. doi: 10.1080/1744666X.2021.1912596 PMC8127172 33792473

[7] Zhou F Yu T Du R Fan G Liu Y Liu Z. Clinical Course and Risk Factors for Mortality of Adult Inpatients With COVID-19 in Wuhan, China: A Retrospective Cohort Study. Lancet (Lond Engl) (2020) 395(10229):1054–62. doi: 10.1016/S0140-6736(20)30566-3 PMC7270627 32171076

[8] Weiss P Murdoch DR. Clinical Course and Mortality Risk of Severe COVID-19. Lancet (Lond Engl) (2020) 395(10229):1014–5. doi: 10.1016/S0140-6736(20)30633-4 PMC7138151 32197108

[9] Gao Y Chen Y Liu M Shi S Tian J. Impacts of Immunosuppression and Immunodeficiency on COVID-19: A Systematic Review and Meta-Analysis. J Infect (2020) 81(2):e93–5. doi: 10.1016/j.jinf.2020.05.017 PMC7228685 32417309

[10] Abedi V Olulana O Avula V Chaudhary D Khan A Shahjouei S. Racial, Economic, and Health Inequality and COVID-19 Infection in the United States. J Racial Ethnic Health Disparities (2021) 8(3):732–42. doi: 10.1007/s40615-020-00833-4 PMC7462354 32875535

[11] Magesh S John D Li WT Li Y Mattingly-App A Jain S. Disparities in COVID-19 Outcomes by Race, Ethnicity, and Socioeconomic Status: A Systematic-Review and Meta-Analysis. JAMA Netw Open (2021) 4(11):e2134147. doi: 10.1001/jamanetworkopen.2021.34147 34762110 PMC8586903

[12] Meyts I Bucciol G Quinti I Neven B Fischer A Seoane E. Coronavirus Disease 2019 in Patients With Inborn Errors of Immunity: An International Study. J Allergy Clin Immunol (2021) 147(2):520–31. doi: 10.1016/j.jaci.2020.09.010 PMC7832563 32980424

[13] Shields AM Burns SO Savic S Richter AG Consortium UPC. COVID-19 in Patients With Primary and Secondary Immunodeficiency: The United Kingdom Experience. J Allergy Clin Immunol (2021) 147(3):870–5. doi: 10.1016/j.jaci.2020.12.620 PMC7737531 33338534

[14] Esenboga S Ocak M Akarsu A Bildik HN Cagdas D Iskit AT. COVID-19 in Patients With Primary Immunodeficiency. J Clin Immunol (2021) 41(7):1515–22. doi: 10.1007/s10875-021-01065-9 PMC8260154 34231093

[15] Bastard P Rosen LB Zhang Q Michailidis E Hoffmann HH Zhang Y. Auto-Antibodies Against Type I IFNs in Patients With Life-Threatening COVID-19. Science (NYNY) (2020) 218(4):e20202486. doi: 10.1084/jem.20202486 PMC7857397 32972996

[16] Zhang QA Bastard PA Liu Z Le Pen JA Moncada-Velez MA Chen J. Inborn Errors of Type I IFN Immunity in Patients With Life-Threatening COVID-19. Science (2020) 370(6515):eabd4585. doi: 10.1126/science.abd4585 32972995 PMC7857407

[17] Quinti I Lougaris V Milito C Cinetto F Pecoraro A Mezzaroma I. A Possible Role for B Cells in COVID-19? Lesson From Patients With Agammaglobulinemia. J Allergy Clin Immunol (2020) 146(1):1–213.e4. doi: 10.1016/j.jaci.2020.04.013 32333914 PMC7175894

[18] Jin H Reed JC Liu STH Ho HE Lopes JP Ramsey NB. Three Patients With X-Linked Agammaglobulinemia Hospitalized for COVID-19 Improved With Convalescent Plasma. J Allergy Clin Immunol In Pract (2020) 8(10):3594–6.e3. doi: 10.1016/j.jaip.2020.08.059 32947026 PMC7490621

[19] Mira E Yarce OA Ortega C Fernandez S Pascual NM Gomez C. Rapid Recovery of a SARS-CoV-2-Infected X-Linked Agammaglobulinemia Patient After Infusion of COVID-19 Convalescent Plasma. J Allergy Clin Immunol In Pract (2020) 8(8):2793–5. doi: 10.1016/j.jaip.2020.06.046 PMC7345404 32652231

[20] Van Damme KFA Tavernier S Van Roy N De Leeuw E Declercq J Bosteels C. Case Report: Convalescent Plasma, a Targeted Therapy for Patients With CVID and Severe COVID-19. Front Immunol (2020) 11(1664-3224:596761). doi: 10.3389/fimmu.2020.596761 33329586 PMC7714937

[21] Ponsford MJ Shillitoe BMJ Humphreys IR Gennery AR Jolles S. COVID-19 and X-Linked Agammaglobulinemia (XLA) - Insights From a Monogenic Antibody Deficiency. Curr Opin Allergy Clin Immunol (2021) 21(6):525–34. doi: 10.1097/ACI.0000000000000792 34596095

[22] Grammatikos A Donati M Johnston SL Gompels MM. Peripheral B Cell Deficiency and Predisposition to Viral Infections: The Paradigm of Immune Deficiencies. Front Immunol (2021) 12(1664–3224):731643. doi: 10.3389/fimmu.2021.731643 34527001 PMC8435594

[23] Hensley MK Bain WG Jacobs J Nambulli S Parikh U Cillo A. Intractable Coronavirus Disease 2019 (COVID-19) and Prolonged Severe Acute Respiratory Syndrome Coronavirus 2 (SARS-CoV-2) Replication in a Chimeric Antigen Receptor-Modified T-Cell Therapy Recipient: A Case Study. Clin Infect Dis (2021) 73(3):e815–21. doi: 10.1093/cid/ciab072 PMC7929077 33507235

[24] Chen L Zody MC Di Germanio C Martinelli R Mediavilla JR Cunningham MH. Emergence of Multiple SARS-CoV-2 Antibody Escape Variants in an Immunocompromised Host Undergoing Convalescent Plasma Treatment. mSphere (2021) 6(4):e0048021. doi: 10.1128/mSphere.00480-21 34431691 PMC8386433

[25] Bertoletti A Le Bert N Qui M Tan AT. SARS-CoV-2-Specific T Cells in Infection and Vaccination. Cell Mol Immunol (2021) 18(10):2307–12. doi: 10.1038/s41423-021-00743-3 PMC8408362 34471260

[26] Dash GC Subhadra S Turuk J Parai D Rath S Sabat J. Breakthrough SARS-CoV-2 Infections Among BBV-152 (COVAXIN^®^) and AZD1222 (COVISHIELDT(M)) Recipients: Report From Eastern State of India. J Med Virol (2021) 0.1002/jmv.27382. doi: 10.1002/jmv.27382 PMC8661601 34622961

[27] Faggiano F Rossi MA Cena T Milano F Barale A Ristagno Q. An Outbreak of COVID-19 Among mRNA-Vaccinated Nursing Home Residents. Vaccines (Basel) 9(8):859. doi: 10.3390/vaccines9080859 34451984 PMC8402388

[28] Bergwerk M Gonen T Lustig Y Amit S Lipsitch M Cohen C. Covid-19 Breakthrough Infections in Vaccinated Health Care Workers. N Engl J Med (2021) 385(16):1474–84. doi: 10.1056/NEJMoa2109072 PMC8362591 34320281

[29] Pegu AA O’Connell S Schmidt SA O'Dell S Talana CA Lai L. Durability of mRNA-1273 Vaccine-Induced Antibodies Against SARS-CoV-2 Variants. Science 373(6561):1372–7. doi: 10.1126/science.abj4176 PMC8691522 34385356

[30] Sahin U Muik A Vogler I Derhovanessian E Kranz LM Vormehr M. BNT162b2 Vaccine Induces Neutralizing Antibodies and Poly-Specific T Cells in Humans. Nature (2021) 595(7868):572–7. doi: 10.1038/s41586-021-03653-6 34044428

[31] Ameratunga R Woon ST Jordan A Longhurst H Leung E Steele R. Perspective: Diagnostic Laboratories Should Urgently Develop T Cell Assays for SARS-CoV-2 Infection. Expert Rev Clin Immunol (2021) 17(5):421–30. doi: 10.1080/1744666X.2021.1905525 33745411

[32] Cevik M Grubaugh ND Iwasaki A Openshaw P. COVID-19 Vaccines: Keeping Pace With SARS-CoV-2 Variants. Cell (2021) 184(20):5077–81. doi: 10.1016/j.cell.2021.09.010 PMC8445744 34534444

[33] Ameratunga R Woon ST. Perspective: Evolving Concepts in the Diagnosis and Understanding of Common Variable Immunodeficiency Disorders (CVID). Clin Rev Allergy Immunol (2020) 59(1):109–21. doi: 10.1007/s12016-019-08765-6 31720921

[34] Ameratunga R Allan C Woon ST. Defining Common Variable Immunodeficiency Disorders in 2020. Immunol Allergy Clin N Am (2020) 40(3):403–20. doi: 10.1016/j.iac.2020.03.001 32654689

[35] Ameratunga R Woon ST Gillis D Koopmans W Steele R. New Diagnostic Criteria for Common Variable Immune Deficiency (CVID), Which May Assist With Decisions to Treat With Intravenous or Subcutaneous Immunoglobulin. Clin Exp Immunol (2013) 174(2):203–11. doi: 10.1111/cei.12178 PMC3828823 23859429

[36] Seidel MG Kindle G Gathmann B Quinti I Buckland M van Montfrans J. The European Society for Immunodeficiencies (ESID) Registry Working Definitions for the Clinical Diagnosis of Inborn Errors of Immunity. J Allergy Clin Immunol In Pract (2019) 7(6):1763–70. doi: 10.1016/j.jaip.2019.02.004 30776527

[37] Bonilla FA Barlan I Chapel H Costa-Carvalho BT Cunningham-Rundles C de la Morena MT. International Consensus Document (ICON): Common Variable Immunodeficiency Disorders. J Allergy Clin Immunol In Pract (2016) 4(1):38–59. doi: 10.1016/j.jaip.2015.07.025 26563668 PMC4869529

[38] Ameratunga R Allan C Lehnert K Woon ST. Perspective: Application of the American College of Medical Genetics Variant Interpretation Criteria to Common Variable Immunodeficiency Disorders. Clin Rev Allergy Immunol (2021) 61(2):226–35. doi: 10.1007/s12016-020-08828-z 33818703

[39] Maffucci P Filion CA Boisson B Itan Y Shang L Casanova JL. Genetic Diagnosis Using Whole Exome Sequencing in Common Variable Immunodeficiency. Front Immunol (2016) 7:220. doi: 10.3389/fimmu.2016.00220 27379089 PMC4903998

[40] Edwards ESJ Bosco JJ Ojaimi S O’Hehir RE van Zelm MC. Beyond Monogenetic Rare Variants: Tackling the Low Rate of Genetic Diagnoses in Predominantly Antibody Deficiency. Cell Mol Immunol (2021) 18(3):588–603. doi: 10.1038/s41423-020-00520-8 32801365 PMC8027216

[41] Abolhassani H Aghamohammadi A Fang M Rezaei N Jiang C Liu X. Clinical Implications of Systematic Phenotyping and Exome Sequencing in Patients With Primary Antibody Deficiency. Genet Med (2019) 21(1):243–51. doi: 10.1038/s41436-018-0012-x 29921932

[42] Conley ME Notarangelo LD Etzioni A. Diagnostic Criteria for Primary Immunodeficiencies. Representing PAGID (Pan-American Group for Immunodeficiency) and ESID (European Society for Immunodeficiencies). Clin Immunol (1999) 93(3):190–7. doi: 10.1006/clim.1999.4799 10600329

[43] Seppanen M Aghamohammadi A Rezaei N. Is There a Need to Redefine the Diagnostic Criteria for Common Variable Immunodeficiency? Expert Rev Clin Immunol (2014) 10(1):1–5. doi: 10.1586/1744666X.2014.870478 24325452

[44] Ameratunga R Ahn Y Jordan A Lehnert K Brothers S Woon ST. Keeping It in the Family: The Case for Considering Late-Onset Combined Immunodeficiency a Subset of Common Variable Immunodeficiency Disorders. Expert Rev Clin Immunol (2018) 14(7):549–56. doi: 10.1080/1744666X.2018.1481750 29806948

[45] Fliegauf M Bryant VL Frede N Slade C Woon ST Lehnert K. Haploinsufficiency of the NF-Kappa Beta 1 Subunit P50 in Common Variable Immunodeficiency. Am J Hum Genet (2015) 97(3):389–403. doi: 10.1016/j.ajhg.2015.07.008 26279205 PMC4564940

[46] Lorenzini T Fliegauf M Klammer N Frede N Proietti M Bulashevska A. Characterization of the Clinical and Immunological Phenotype and Management of 157 Individuals With 56 Distinct Heterozygous NFKB1 Mutations. J Allergy Clin Immunol (2020) 9(20):051. doi: 10.1016/j.jaci.2019.11.051 PMC8246418 32278790

[47] Ameratunga R Ahn Y Steele R Woon ST. The Natural History of Untreated Primary Hypogammaglobulinemia in Adults: Implications for the Diagnosis and Treatment of Common Variable Immunodeficiency Disorders (CVID). Front Immunol (2019) 10:1541. doi: 10.3389/fimmu.2019.01541 31379811 PMC6652801

[48] Ameratunga R Jordan A Cavadino A Ameratunga S Hills T Steele R. Bronchiectasis Is Associated With Delayed Diagnosis and Adverse Outcomes in the New Zealand Common Variable Immunodeficiency Disorders Cohort Study. Clin Exp Immunol (2021) 204(3):352–60. doi: 10.1111/cei.13595 PMC8119856 33755987

[49] Ameratunga R Longhurst H Steele R Woon ST. Comparison of Diagnostic Criteria for Common Variable Immunodeficiency Disorders (CVID) in the New Zealand CVID Cohort Study. Clin Rev Allergy Immunol (2021) 61(2):236–44. doi: 10.1007/s12016-021-08860-7 34236581

[50] Amodio D Ruggiero A Sgrulletti M Pighi C Cotugno N Medri C. Humoral and Cellular Response Following Vaccination With the BNT162b2 mRNA COVID-19 Vaccine in Patients Affected by Primary Immunodeficiencies. Front Immunol (2021) 12(1664–3224). doi: 10.3389/fimmu.2021.727850 PMC8521226 34671350

[51] Salinas AF Mortari EP Terreri S Quintarelli C Pulvirenti F Di Cecca S. SARS-CoV-2 Vaccine Induced Atypical Immune Responses in Antibody Defects: Everybody Does Their Best. J Clin Immunol (2021) 41(8):1709–22. doi: 10.1007/s10875-021-01133-0 PMC8527979 34669144

[52] Ameratunga R Longhurst H Steele R Lehnert K Leung E Brooks AES. Common Variable Immunodeficiency Disorders, T Cell Responses to SARS-CoV-2 Vaccines and the Risk of Chronic COVID-19. J Allergy Clin Immunol Pract (2021) 9(10):3575–83. doi: 10.1016/j.jaip.2021.06.019 PMC8230758 34182162

[53] Felten RA Duret PM Bauer E Sedmak N Djossou JH Bensalem M. B-Cell Targeted Therapy Is Associated With Severe COVID-19 Among Patients With Inflammatory Arthritides: A 1-Year Multicentre Study in 1116 Successive Patients Receiving Intravenous Biologics. Ann Rheum Dis (2021) annrheumdis-2021-220549. doi: 10.1136/annrheumdis-2021-220549 34556483

[54] Levavi HA-O Lancman GA Gabrilove JA. Impact of Rituximab on COVID-19 Outcomes. Ann Hematol (2021) 100(11):2805–12. doi: 10.1007/s00277-021-04662-1 PMC8455155 34549309

[55] Marasco V Carniti CA Guidetti A Farina L Magni M Miceli R. T-Cell Immune Response After mRNA SARS-CoV-2 Vaccines Is Frequently Detected Also in the Absence of Seroconversion in Patients With Lymphoid Malignancies. Br J Haematol (2021) 10.1111/bjh.17877. doi: 10.1111/bjh.17877 PMC8653177 34649298

[56] Hall VG Ferreira VH Ku T Ierullo M Majchrzak-Kita B Chaparro C. Randomized Trial of a Third Dose of mRNA-1273 Vaccine in Transplant Recipients. N Engl J Med (2021) 385(13):1244–6. doi: 10.1056/NEJMc2111462 PMC8385563 34379917

[57] Shroff RT Chalasani P Wei R Pennington D Quirk G Schoenle MV. Immune Responses to Two and Three Doses of the BNT162b2 mRNA Vaccine in Adults With Solid Tumors. Nat Med (2021) 27(11):2002–11. doi: 10.1038/s41591-021-01542-z PMC9004706 34594036

[58] Benning LA Töllner M Hidmark A Schaier MA Nusshag CA Kälble F. Heterologous ChAdOx1 nCoV-19/BNT162b2 Prime-Boost Vaccination Induces Strong Humoral Responses Among Health Care Workers. Vaccines (Basel) 9(8):857. doi: 10.3390/vaccines9080857 34451982 PMC8402499

[59] Dodd RA Notari EP Brodsky JP Foster GA Xu M Saá P. Patterns of Antibody Response to SARS-CoV-2 Among 1.6 Million Blood Donors: Impact of Vaccination, United States December 2020 - June 2021. J Infect Dis (2021) jiab514. doi: 10.1093/infdis/jiab514 34626465

[60] Hartley GA Edwards EA Bosco JA Ojaimi SA Stirling RA Cameron PA. Influenza-Specific IgG1(+) Memory B-Cell Numbers Increase Upon Booster Vaccination in Healthy Adults But Not in Patients With Predominantly Antibody Deficiency. Clin Transl Immunol (2020) 9(10):e1199. doi: 10.1002/cti2.1199 PMC7563650 33088507

[61] Ameratunga RA Woon ST Jordan A Longhurst H Leung EA Steele R. Response to Letter to the Editor: The Clinical Utility of Diagnostic T Cell Assays for COVID-19. Expert Rev Clin Immunol (2021) 17(11):1159–61. doi: 10.1080/1744666X.2021.1982386 PMC8544663 34530670

